# Supplementary material for: Immune signature of acute pharyngitis in a Streptococcus pyogenes human challenge trial
Source: Nat Commun. 2022 Feb 9;13:769. doi: 10.1038/s41467-022-28335-3 (PMC8828729; doi:10.1038/s41467-022-28335-3)
Supplement: Supplementary file 1 — Supplementary Information [file 41467_2022_28335_MOESM1_ESM.pdf]

Supplementary Information

Immune signature of acute pharyngitis in a *Streptococcus pyogenes* human challenge trial

Jeremy Anderson[1,2], Samira Imran[1,2], Hannah R Frost[1], Kristy I. Azzopardi[1], Sedigheh Jalali[1,2], Boris Novakovic[1,2], Joshua Osowicki[1,2,3*], Andrew C Steer[1,2,3*], Paul V Licciardi[1,2*] Daniel G Pellicci[1,2,4*]

[1]Murdoch Children's Research Institute, Melbourne, Australia, [2]Department of Paediatrics, University of Melbourne, Melbourne, Australia, [3]Infectious Diseases Unit, Department of General Medicine, The Royal Children's Hospital Melbourne, Melbourne, Victoria, Australia, [4]Department of Microbiology and Immunology, University of Melbourne, Melbourne, Australia.

* = contributed equally

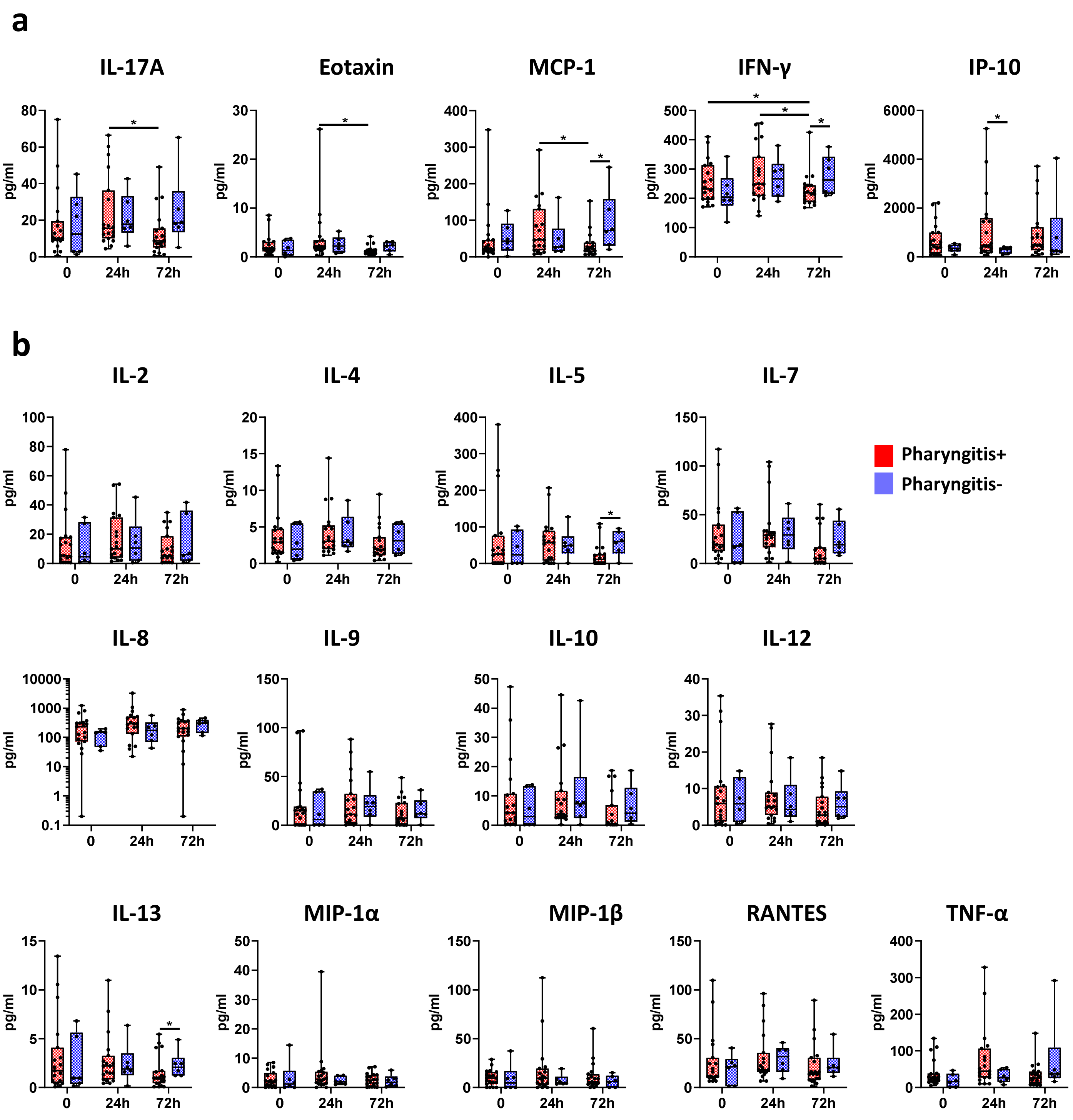

Supplementary Figure 1: Additional saliva cytokines/chemokines analysed. a, Cytokines/chemokines that decreased during infection at timepoint 0h (pre-infection) 24h post-infection (24h) and 72h post-infection (72h) between pharyngitis positive (n = 19, red) and negative (n = 6, blue) participants. **b,** Cytokines/chemokines that did not change during infection. Data presented as boxplots shown median ± IQR with min-max whiskers. A Friedman's test was used to compare responses over-time in P+ and P- groups, whilst a Mann-Whitney U-test was performed to compare differences between P+ and P- groups. All tests performed were two-tailed and a p-value <0.05 was considered significant; *p<0.05, **p<0.01, ***p<0.001, ****p<0.0001.

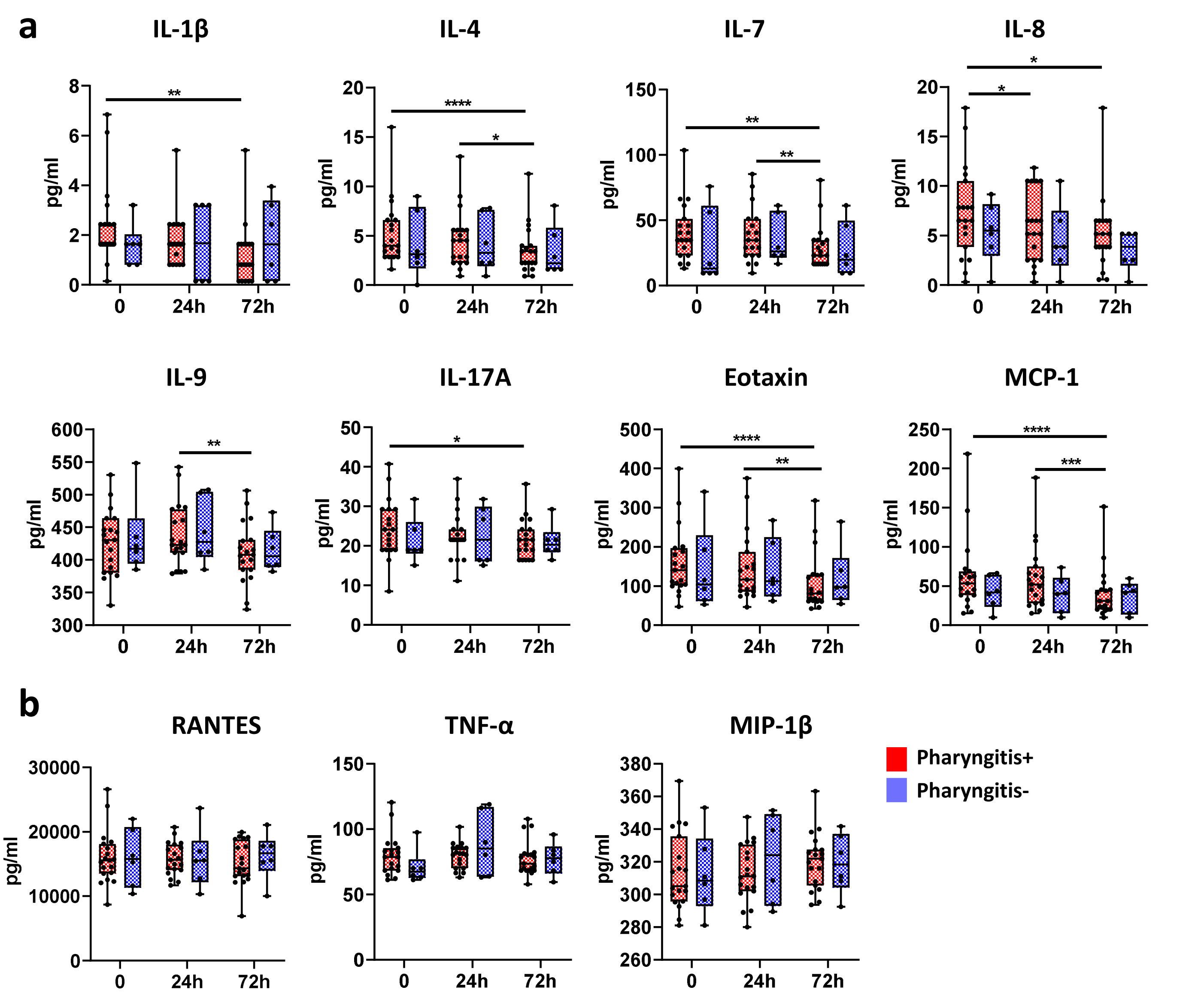

Supplementary Figure 2: Additional serum cytokines/chemokines analysed. a, Cytokines/chemokines that decreased during infection timepoint 0h (pre-infection) 24h post-infection (24h) and 72h post-infection (72h) between pharyngitis positive (n = 19, red) and negative (n = 6, blue) participants. **b,** Cytokines/chemokines that did not change during infection. Data presented as boxplots shown median ± IQR with min-max whiskers. A Friedman's test was used to compare responses over-time in P+ and P- groups, whilst a Mann-Whitney U-test was performed to compare differences between P+ and P- groups. All tests performed were two-tailed and a p-value <0.05 was considered significant; *p<0.05, **p<0.01, ***p<0.001, ****p<0.0001.

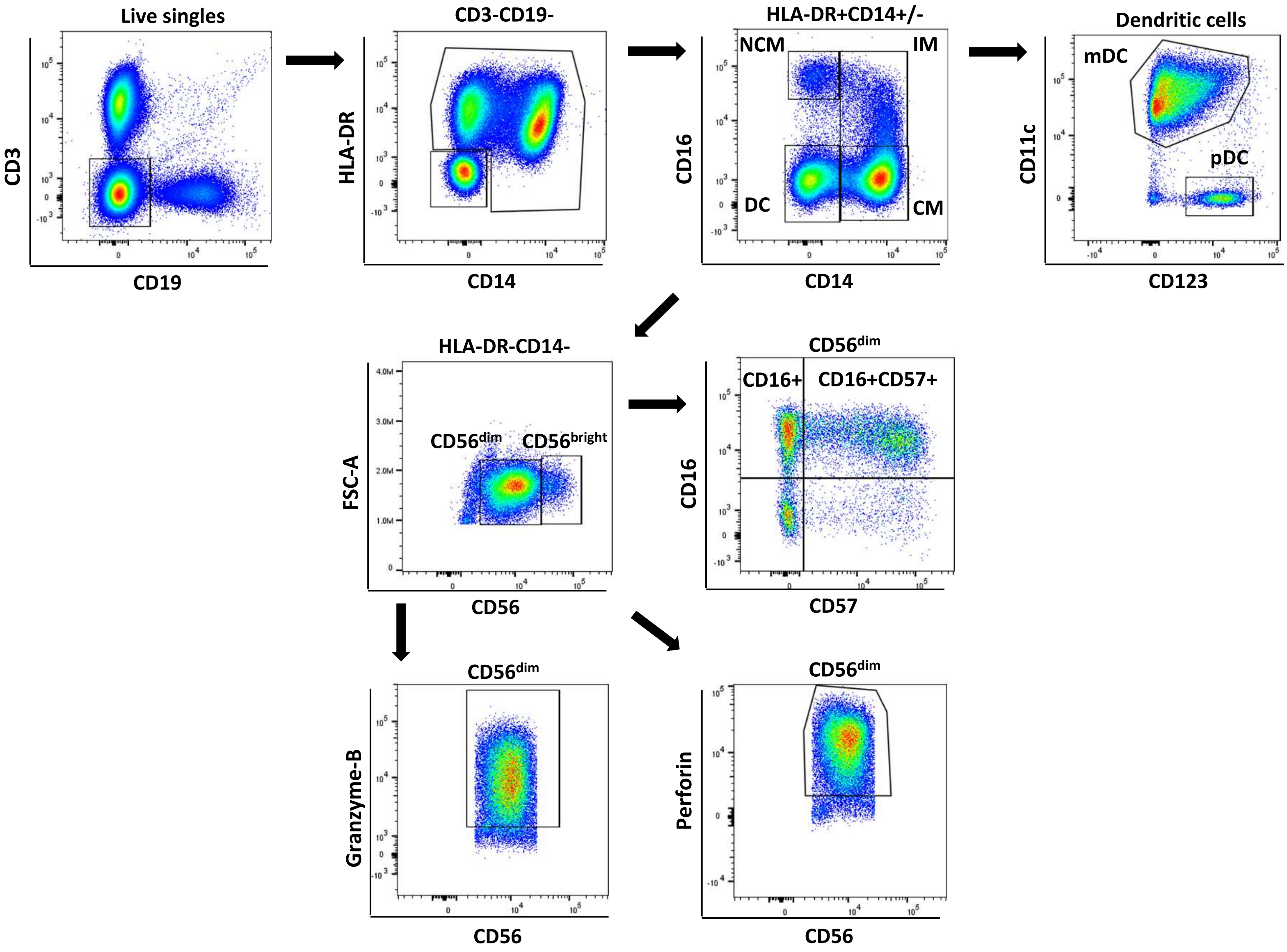

Supplementary Figure 3: Gating strategy to identify innate cell subsets. From live single cells, T and B-cells were removed by gating the CD3-CD19- population. HLA-DR and CD14 were used to discriminate between myeloid cells (HLA-DR+CD14+/-) and NK cells (HLA-DR-CD14-). From the myeloid cell populations, CD16 and CD16 were used to discriminate non-classical monocytes (NCM; CD14-CD16+), intermediate monocytes (IM; CD14+CD16+), classical monocytes (CM; CD14+CD16-) and dendritic cells (DC; CD14-CD16-). Dendritic cells were further categorised into myeloid dendritic cells (mDCs, CD11c+CD123-) and plasmacytoid dendritic cells (pDCs; CD11c-CD123+). NK cells were divided into $CD56^{dim}$ and $CD56^{bright}$. From the $CD56^{dim}$ population, CD16, CD57, perforin and granzyme B positive populations were identified.

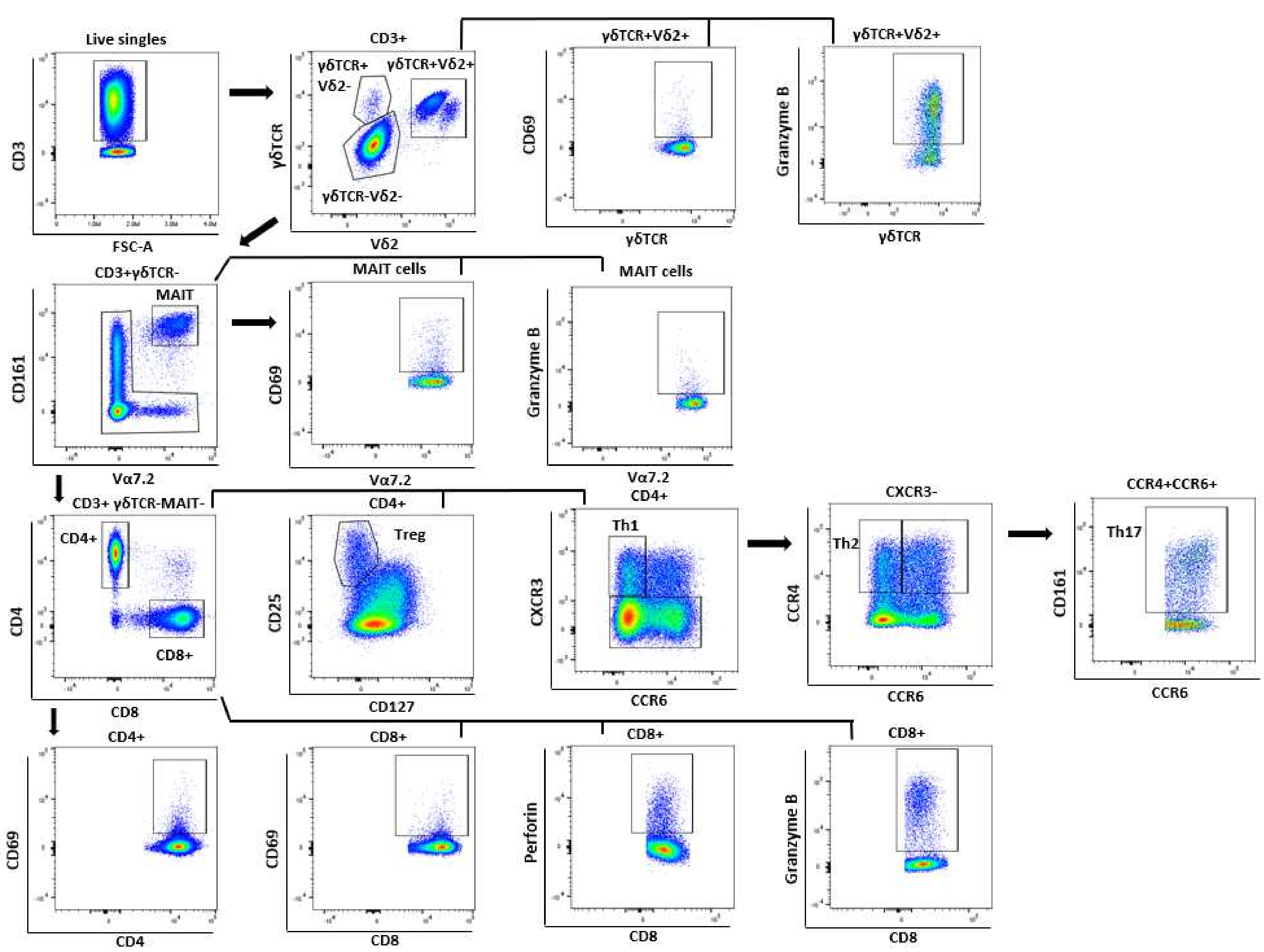

Supplementary Figure 4: Gating strategy to identify T-cell subsets. From live single cells, T-cells were identified by positive CD3 expression. γδTCR+ and γδTCR+Vδ2+ T-cells were identified and CD69+ and granzyme B+ expression was characterised on the γδTCR+Vδ2+ population. From the γδTCR- population, MAIT cells were identified by CD161+Vα7.2+ expression and CD69+ and granzyme B+ expression was characterised on the MAIT cell population. From the MAIT cell negative population, CD4+ and CD8+ T-cells were identified. CD4+ T-cells were characterised into Treg (CD25+CD127^{lo}), Th1 (CXCR3+), Th2 (CXCR3-CCR4+CCR6-) and Th17 (CXCR3-CCR4+CCR6+CD161+). CD69+ expression was also explored on CD4+ T-cells. CD8+ T-cells were further characterised into CD69, perforin and granzyme B expression CD8+ T-cells.

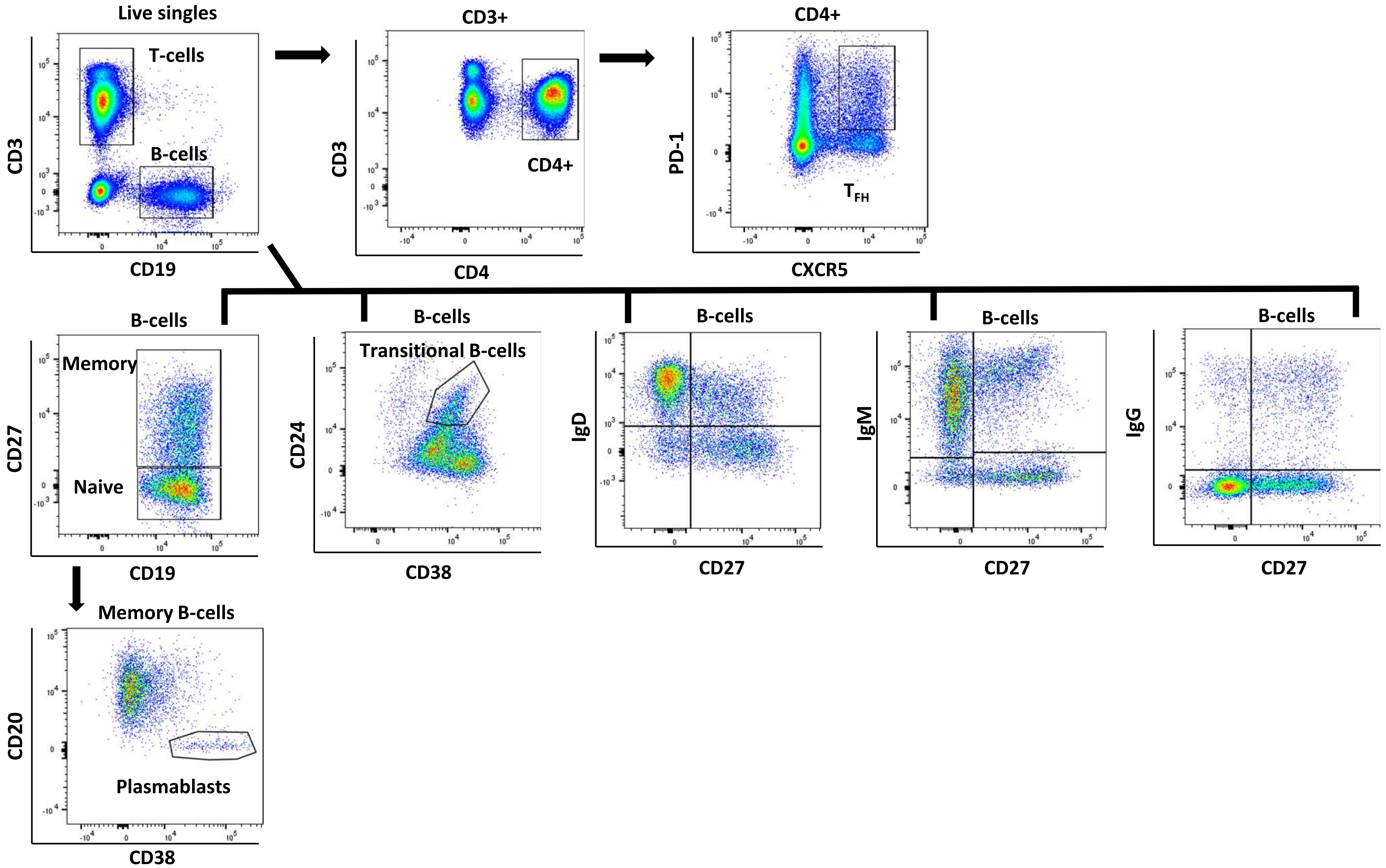

Supplementary Figure 5: Gating strategy to identify B-cell/T_{FH} subsets. From live single cells, T-cells were identified as CD3+CD19- and B-cells were identified as the CD3-CD19+ population. From the CD3+ population, T_{FH} was characterised as CD4+CXCR5+PD-1+ expressing T-cells. From the B-cell population, memory was identified as CD27+, transitional B-cells as CD24+CD38+ and plasmablasts as CD27+CD20-CD38+. IgD, IgM and IgG expression were also characterised on B-cell populations.

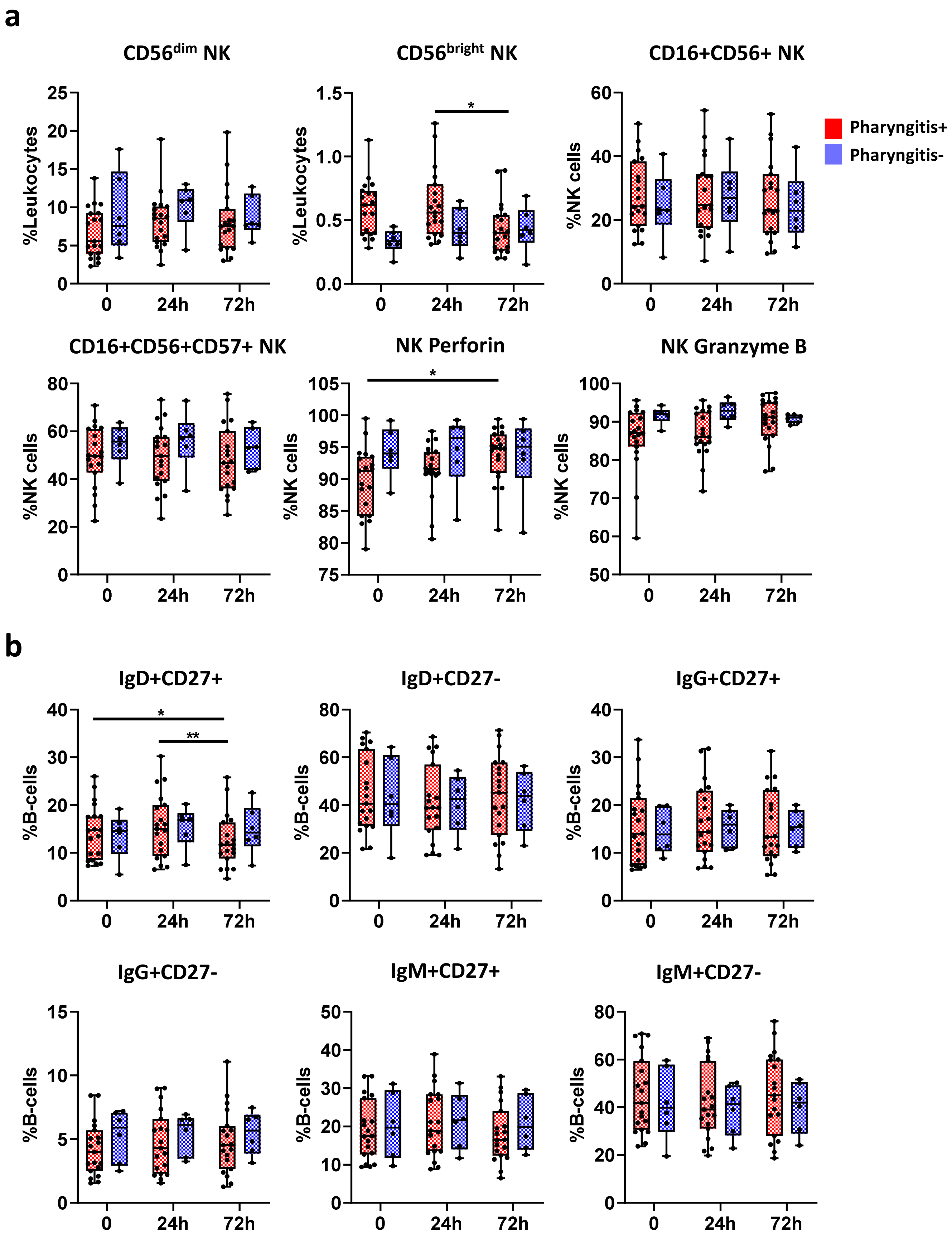

Supplementary Figure 6: Additional innate and B-cell subsets analysed. a, NK cell subsets at timepoint 0 (pre-infection) 24h post-infection (24h) and 72h post-infection (72h) between pharyngitis positive (n = 19, red) and negative (n = 6, blue) participants. **b,** B-cells expressing IgD, IgM and IgG. Data presented as boxplots shown median ± IQR with min-max whiskers. A Friedman's test was used to compare responses over-time in P+ and P- groups, whilst a Mann-Whitney U-test was performed to compare differences between P+ and P- groups. All tests performed were two-tailed and a p-value <0.05 was considered significant; *p<0.05, **p<0.01, ***p<0.001, ****p<0.0001.

Supplementary Table 1: Surface staining antibody cocktails

Antibody Cocktail 1	Supplier	Antibody Cocktail 2	Supplier	Antibody Cocktail 3	Supplier
CXCR3-APC	BD Bioscience, San Diego, CA, USA	CD3-BUV737	BD Bioscience, San Diego, CA, USA	CD24-BV711	BioLegend, San Diego, USA
CCR6-BUV496	BD Bioscience, San Diego, CA, USA	CD16-BV605	BD Bioscience, San Diego, CA, USA	HLA-DR-APC	BD Bioscience, San Diego, CA, USA
CCR4-BV605	BioLegend, San Diego, USA	HLA-DR-BUV395	BD Bioscience, San Diego, CA, USA	CD20-BV421	BioLegend, San Diego, USA
γδTCR-FITC	BD Bioscience, San Diego, CA, USA	CD11c-AF647	BioLegend, San Diego, USA	CD27-BUV737	BD Bioscience, San Diego, CA, USA
CD127-APC-R700	BioLegend, San Diego, USA	CD56-BV510	BioLegend, San Diego, USA	IgD-BUV395	BD Bioscience, San Diego, CA, USA
CD25-PE-CF594	BD Bioscience, San Diego, CA, USA	CD14-BUV805	BD Bioscience, San Diego, CA, USA	CD19-BV785	BioLegend, San Diego, USA
Vδ2-BV480	BD Bioscience, San Diego, CA, USA	CD123-PEcy7	BD Bioscience, San Diego, CA, USA	CD38-BUV496	BD Bioscience, San Diego, CA, USA
CD161-PEvio770	Miltenyi Biotec, New South Wales, Australia	CD19-BV785	BioLegend, San Diego, USA	CD4-BV510	BioLegend, San Diego, USA
CD3-BUV395	BD Bioscience, San Diego, CA, USA	CD57-PE-CF594	BD Bioscience, San Diego, CA, USA	CD3-PerCP/Cy5.5	BD Bioscience, San Diego, CA, USA
CD4-BV421	BD Bioscience, San Diego, CA, USA	Zombie NIR	BioLegend, San Diego, USA	CXCR5-APCR700	BD Bioscience, San Diego, CA, USA
CD8-BUV805	BD Bioscience, San Diego, CA, USA			PD-1-PEcy7	BD Bioscience, San Diego, CA, USA
CD69-BV650	BioLegend, San Diego, USA			IgG-BV605	BD Bioscience, San Diego, CA, USA
Vα7.2-BV711	BioLegend, San Diego, USA			IgM-FITC	BioLegend, San Diego, USA
Zombie NIR	BioLegend, San Diego, USA			Zombie NIR	BioLegend, San Diego, USA

Supplementary Table 2: Intracellular antibody cocktails

Antibody Cocktail 1	Supplier
Granzyme B-Pacific Blue	BioLegend, San Diego, USA
Perforin-APCcy7	BioLegend, San Diego, USA
