# Supplementary material for: Immune signature of acute pharyngitis in a Streptococcus pyogenes human challenge trial
Source: Nat Commun. 2022 Feb 9;13:769. doi: 10.1038/s41467-022-28335-3 (PMC8828729; doi:10.1038/s41467-022-28335-3)
Supplement: Supplementary file 3 — Reporting Summary [file 41467_2022_28335_MOESM3_ESM.pdf]

nature portfolio

Corresponding author(s): Daniel G Pellicci
Paul V Licciardi
Andrew Steer
Josh Osowicki

Last updated by author(s): Jan17th2022

Reporting Summary

Nature Portfolio wishes to improve the reproducibility of the work that we publish. This form provides structure for consistency and transparency in reporting. For further information on Nature Portfolio policies, see our Editorial Policies and the Editorial Policy Checklist.

Statistics

For all statistical analyses, confirm that the following items are present in the figure legend, table legend, main text, or Methods section.

n/a	Confirmed	
☐	☒	The exact sample size (n) for each experimental group/condition, given as a discrete number and unit of measurement
☐	☒	A statement on whether measurements were taken from distinct samples or whether the same sample was measured repeatedly
☐	☒	The statistical test(s) used AND whether they are one- or two-sided<br>*Only common tests should be described solely by name; describe more complex techniques in the Methods section.*
☐	☒	A description of all covariates tested
☐	☒	A description of any assumptions or corrections, such as tests of normality and adjustment for multiple comparisons
☐	☒	A full description of the statistical parameters including central tendency (e.g. means) or other basic estimates (e.g. regression coefficient) AND variation (e.g. standard deviation) or associated estimates of uncertainty (e.g. confidence intervals)
☐	☒	For null hypothesis testing, the test statistic (e.g. F, t, r) with confidence intervals, effect sizes, degrees of freedom and P value noted<br>*Give P values as exact values whenever suitable.*
☒	☐	For Bayesian analysis, information on the choice of priors and Markov chain Monte Carlo settings
☒	☐	For hierarchical and complex designs, identification of the appropriate level for tests and full reporting of outcomes
☐	☒	Estimates of effect sizes (e.g. Cohen's d, Pearson's r), indicating how they were calculated

Our web collection on statistics for biologists contains articles on many of the points above.

Software and code

Policy information about availability of computer code

Data collection	Cytek Aurora, Luminex 200 instrument, Bio-Plex Manager Version 6 software
Data analysis	Flowjo Vl0, UMAP plugin within Flowjo VlO, Prism V8, R v3.6. 1

For manuscripts utilizing custom algorithms or software that are central to the research but not yet described in published literature, software must be made available to editors and reviewers. We strongly encourage code deposition in a community repository (e.g. GitHub). See the Nature Portfolio guidelines for submitting code & software for further information.

Data

Policy information about availability of data

All manuscripts must include a data availability statement. This statement should provide the following information, where applicable:

Most data generated or analysed during this study are included in this published article (and its supplementary information files). Any additional data are available from the corresponding authors upon reasonable request.

- Accession codes, unique identifiers, or web links for publicly available datasets
- A description of any restrictions on data availability
- For clinical datasets or third party data, please ensure that the statement adheres to our policy

The source data underlying all figures and supplemental data will be provided as a Source Data file

Field-specific reporting

Please select the one below that is the best fit for your research. If you are not sure, read the appropriate sections before making your selection.

- [x] Life sciences
- [ ] Behavioural & social sciences
- [ ] Ecological, evolutionary & environmental sciences

For a reference copy of the document with all sections, see nature.com/documents/nr-reporting-summary-flat.pdf

Life sciences study design

All studies must disclose on these points even when the disclosure is negative.

ample size	The study consists of 25 healthy adults. Samples were collected at 3 time points, pre-infection (0h), post-infection (24h) and post-infection (72h). These were determined with reference to the vaccine efficacy targets in the WHO GAS Vaccines Research and Development Roadmap. https://academic.oup.com/cid/article/69/5/877/5280612 Further details are found in: 'Osowicki, J. et al. A controlled human infection model of Streptococcus pyogenes pharyngitis (CHIVAS-M75): an observational, dose-finding study. The Lancet Microbe (2021)' and 'Osowicki, J. et al. Controlled human infection for vaccination against Streptococcus pyogenes (CHIVAS): Establishing a group A Streptococcus pharyngitis human infection study. Vaccine 37, 3485-3494, doi:10.1016/j.vaccine.2019.03.059 (2019)'.
Data exclusions	No data were excluded
Replication	Sample testing was not repeated due to availability of samples.
andomization	This study was based on comparing participants who did develop pharyngitis after challenge (n=19) with those who did not (n=6) in an initial non-randomised dose-finding human challenge trial to establish a new model of S. pyogenes phayngitis.
Blinding	During data collection and analysis for this study, the investigators were not blinded. As this study is a follow-up to the previously published primary objective manuscript 'Osowicki, J. et al. A controlled human infection model of Streptococcus pyogenes pharyngitis (CHIVAS-M75): an observational, dose-finding study. The Lancet Microbe (2021)' the investigators were aware of which samples were pharyngitis positive or negative.

Reporting for specific materials, systems and methods

We require information from authors about some types of materials, experimental systems and methods used in many studies. Here, indicate whether each material, system or method listed is relevant to your study. If you are not sure if a list item applies to your research, read the appropriate section before selecting a response.

Materials & experimental systems

n/a	Involved in the study	
[ ]	[x]	Antibodies
[x]	[ ]	Eukaryotic cell lines
[x]	[ ]	Palaeontology and archaeology
[x]	[ ]	Animals and other organisms
[ ]	[x]	Human research participants
[ ]	[x]	Clinical data
[x]	[ ]	Dual use research of concern

Methods

n/a	Involved in the study	
[x]	[ ]	ChIP-seq
[ ]	[x]	Flow cytometry
[x]	[ ]	MRI-based neuroimaging

Antibodies

Antibodies used

CXCR3-APC 1/25 BD Bioscience, San Diego, CA, USA 550967
CCR6-BUV496 1/100 BD Bioscience, San Diego, CA, USA 612948
CCR4-BV605 1/100 BioLegend, San Diego, USA 359418
γδTCR-FITC 1/50 BD Bioscience, San Diego, CA, USA 347903
CD127-APC-R700 1/100 BioLegend, San Diego, USA 565185
CD25-PE-CF594 1/100 BD Bioscience, San Diego, CA, USA 562403
Vδ2-BV480 1/400 BD Bioscience, San Diego, CA, USA 746567
CD161-PEvio770 1/200 Miltenyi Biotec, New South Wales, Australia 130-113-594
CD3-BUV395 1/100 BD Bioscience, San Diego, CA, USA 563546
CD4-BV421 1/200 BD Bioscience, San Diego, CA, USA 562424
CD8-BUV805 1/200 BD Bioscience, San Diego, CA, USA 612889
CD69-BV650 1/200 BioLegend, San Diego, USA 310934
Vα7.2-BV711 1/100 BioLegend, San Diego, USA 351732
CD3-BUV737 1/100 BD Bioscience, San Diego, CA, USA 564307
CD16-BV605 1/100 BD Bioscience, San Diego, CA, USA 563172
HLA-DR-BUV395 1/100 BD Bioscience, San Diego, CA, USA 740302
CD11c-AF647 1/200 BioLegend, San Diego, USA 301620
CD56-BV510 1/50 BioLegend, San Diego, USA 318340
CD14-BUV805 1/100 BD Bioscience, San Diego, CA, USA 612902
CD123-PEcy7 1/200 BD Bioscience, San Diego, CA, USA 560826

CD19-BV785 1/400 BioLegend, San Diego, USA 302240
CD57-PE-CF594 1/400 BD Bioscience, San Diego, CA, USA 562488
CD24-BV711 1/200 BioLegend, San Diego, USA 311135
HLA-DR-APC 1/100 BD Bioscience, San Diego, CA, USA 559866
CD20-BV421 1/100 BioLegend, San Diego, USA 302329
CD27-BUV737 1/200 BD Bioscience, San Diego, CA, USA 612829
IgD-BUV395 1/200 BD Bioscience, San Diego, CA, USA 563813
CD19-BV785 1/400 BioLegend, San Diego, USA 302240
CD38-BUV496 1/200 BD Bioscience, San Diego, CA, USA 612946
CD4-BV510 1/200 BioLegend, San Diego, USA 344633
CXCR5-APCR700 1/50 BD Bioscience, San Diego, CA, USA 565191
PD-1-PEcy7 1/50 BD Bioscience, San Diego, CA, USA 561272
IgG-BV605 1/200 BD Bioscience, San Diego, CA, USA 563246
IgM-FITC 1/200 BioLegend, San Diego, USA 314506
CD3 PerCP-Cy5.5 1/100, BD Bioscience, San Diego, CA, USA 560835
Zombie NIR 1/800 BioLegend, San Diego, USA 423106

Validation

All antibodies are mouse or rat anti-human and were titrated on human PBMCs and analysed by flow cytometry. Immune cells known to be negative for a specific antibody were used as negative controls.

Human research participants

Policy information about studies involving human research participants

Population characteristics

25 healthy adults, 12 females and 13 males, mean age 27.6 years, 19 with pharyngitis and 6 without,

Recruitment

Recruitment of participants was originally described in "Osowicki, J. et al. A controlled human infection model of Streptococcus pyogenes pharyngitis (CHIVAS-M75): an observational, dose-finding study. The Lancet Microbe (2021)". This paper is cited within the current manuscript.

Ethics oversight

Alfred Hospital Human Research Ethics Committee (500/17).

Note that full information on the approval of the study protocol must also be provided in the manuscript.

Clinical data

Policy information about clinical studies

All manuscripts should comply with the ICMJEguidelines for publication of clinical research and a completedCONSORT checklist must be included with all submissions.

Clinical trial registration

ClinicalTrials.gov, NCT03361163.

Study protocol

Included in the supplementary appendix of "Osowicki, J. et al. A controlled human infection model of Streptococcus pyogenes pharyngitis (CHIVAS-M75): an observational, dose-finding study. The Lancet Microbe (2021)". https://doi.org/10.1016/S2666-5247(20)30240-8

Data collection

Participants challenged between July 10, 2018 (first participant, first dose), and Sept 23, 2019 in Melbourne, Australia.

Outcomes

Cellular responses were measured by flow cytometry. Cytokine responses were measured by biorad multiplex assay or ELISA. Please refer to methods. The primary outcome of acute symptomatic Streptococcus pyogenes pharyngitis in the clinical trial was assessed according to a pharyngitis case definition including clinical and microbiological criteria, considered from challenge with Streptococcus pyogenes applied directly by swab to the pharynx until diagnosis of pharyngitis or until 5 days after challenge in those who did not develop pharyngitis. The secondary outcomes were broadly defined in descriptive terms, comparing results for trial volunteers who did or did not develop pharyngitis after challenge using a broad range of immunology techniques applied to systemic and mucosal samples collected at multiple timepoints. Further description can be found in: 'Osowicki, J. et al. A controlled human infection model of Streptococcus pyogenes pharyngitis (CHIVAS-M75): an observational, dose-finding study. The Lancet Microbe (2021)' and 'Osowicki, J. et al. Controlled human infection for vaccination against Streptococcus pyogenes (CHIVAS): Establishing a group A Streptococcus pharyngitis human infection study. Vaccine 37, 3485-3494, doi:10.1016/j.vaccine.2019.03.059 (2019)'.

Flow Cytometry

Plots

Confirm that:

- [x] The axis labels state the marker and fluorochrome used (e.g. CD4-FITC).
- [x] The axis scales are clearly visible. Include numbers along axes only for bottom left plot of group (a 'group' is an analysis of identical markers).
- [x] All plots are contour plots with outliers or pseudocolor plots.
- [x] A numerical value for number of cells or percentage (with statistics) is provided.

Methodology

Sample preparation

Cryopreserved PBMCs were thawed at 37°C then washed with 10ml R10 media (RPMI-1640 medium supplemented with 10% FBS, 2mM L-glutamine, 1000IU penicillin-streptomycin) and centrifuged at 400 x g for 5 minutes. PBMCs were washed with 5ml PBS and centrifuged at 400 x g for 5 minutes then blocked (50µl of 1% human FC-block and 10% normal rat serum in PBS) for 20 minutes on ice. PBMCs were then washed with 1ml FACS buffer (PBS supplemented with 2% FBS and 2mM EDTA) and stained with 50µl of antibody cocktail 1, 2 or 3 (Supplementary Table 1) for 20 minutes on ice. PBMCs were washed in 1ml of

FACS buffer then resuspended in 100µl of fixation buffer (BD, Bioscience, San Diego, CA, USA) and incubated on ice for 20 minutes. Following, PBMCs were washed twice in permeabilisation buffer prior to intracellular antibody staining. PBMCs were stained for 30 minutes on ice, washed then resuspended in 100µl FACS buffer for acquisition using the Cytek Aurora. Compensation was performed at the time of acquisition using compensation beads. Data was analysed using Flowjo v10.7.1 software. UMAP was generated using the UMAP FlowJo plugin and 5000 events per sample were concatenated. Gating strategies are shown in Supplementary Figure 3, 4, 5.

Instrument

Cytek Aurora

Software

FlowJo V10

Cell population abundance

No cell populations were sorted.

Gating strategy

Innate cells:
From live single cells, T and B-cells were removed by gating the CD3-CD19- population. HLA-DR and CD14 were used to discriminate between myeloid cells (HLA-DR+CD14+/-) and NK cells (HLA-DR-CD14-). From the myeloid cell populations, CD16 and CD16 were used to discriminate non-classical monocytes (NCM; CD14-CD16+), intermediate monocytes (IM; CD14+CD16+), classical monocytes (CM; CD14+CD16-) and dendritic cells (DC; CD14-CD16-). Dendritic cells were further categorised into myeloid dendritic cells (mDCs, CD11c+CD123-) and plasmacytoid dendritic cells (pDCs; CD11c-CD123+). NK cells were divided into CD56dim and CD56bright. From the CD56dim population, CD16, CD57, perforin and granzyme B positive populations were identified.

T-cells:
From live single cells, T-cells were identified by positive CD3 expression. γδTCR+ and γδTCR+Vδ2+ T-cells were identified and CD69+ and granzyme B+ expression was characterised on the γδTCR+Vδ2+ population. From the γδTCR- population, MAIT cells were identified by CD161+Vα7.2+ expression and CD69+ and granzyme B+ expression was characterised on the MAIT cell population. From the MAIT cell negative population, CD4+ and CD8+ T-cells were identified. CD4+ T-cells were characterised into Treg (CD25+CD127lo), Th1 (CXCR3+), Th2 (CXCR3-CCR4+CCR6-) and Th17 (CXCR3-CCR4+CCR6+CD161+). CD69+ expression was also explored on CD4+ T-cells. CD8+ T-cells were further characterised into CD69, perforin and granzyme B expression CD8+ T-cells.

B-cells/Tfh:
From live single cells, T-cells were identified as CD3+CD19- and B-cells were identified as the CD3-CD19+ population. From the CD3+ population, TFH was characterised as CD4+CXCR5+PD-1+ expressing T-cells. From the B-cell population, memory was identified as CD27+, transitional B-cells as CD24+CD38+ and plasmablasts as CD27+CD20-CD38+. IgD, IgM and IgG expression were also characterised on B-cell populations.

☒ Tick this box to confirm that a figure exemplifying the gating strategy is provided in the Supplementary Information.
